# Engineered extracellular vesicles for ischemic stroke: a systematic review and meta-analysis of preclinical studies

**DOI:** 10.1186/s12951-023-02114-8

**Published:** 2023-10-31

**Authors:** Pengtao Li, Rui Yin, Yihao Chen, Jianbo Chang, Lang Yang, Xiaoyu Liu, Houshi Xu, Xiao Zhang, Shihua Wang, Qin Han, Junji Wei

**Affiliations:** 1 Department of Neurosurgery, Peking Union Medical College Hospital, Peking Union Medical College, Chinese Academy of Medical Sciences, Beijing, China; 2 Institute of Basic Medical Sciences Chinese Academy of Medical Sciences, School of Basic Medicine Peking Union Medical College, Beijing, China

**Keywords:** Stroke, Engineered, Extracellular vesicles, Preclinical study

## Abstract

**Background:**

This systematic review and meta-analysis aimed to evaluate the efficacy of engineered extracellular vesicles (EEVs) in the treatment of ischemic stroke (IS) in preclinical studies and to compare them with natural extracellular vesicles (EVs). The systematic review provides an up-to-date overview of the current state of the literature on the use of EEVs for IS and informs future research in this area.

**Methods:**

We searched PubMed, EMBASE, Web of Science, Cochrane Library, and Scopus databases for peer-reviewed preclinical studies on the therapeutic effect of EEVs on IS.Databases ranged from the inception to August 1, 2023. The outcome measures included infarct volumes, neurological scores, behavioral scores, apoptosis rates, numbers of neurons, and levels of IL-1β, IL-6, and TNF-α. The CAMARADES checklist was used to assess the quality and bias risks of the studies. All statistical analyses were performed using RevMan 5.4 software.

**Results:**

A total of 28 studies involving 1760 animals met the inclusion criteria. The results of the meta-analysis showed that compared to natural EVs, EEVs reduced infarct volume (percentage: SMD = -2.33, 95% CI: -2.92, -1.73; size: SMD = -2.36, 95% CI: -4.09, -0.63), improved neurological scores (mNSS: SMD = -1.78, 95% CI: -2.39, -1.17; Zea Longa: SMD = -2.75, 95% CI: -3.79, -1.71), promoted behavioral recovery (rotarod test: SMD = 2.50, 95% CI: 1.81, 3.18; grid-walking test: SMD = -3.45, 95% CI: -5.15, -1.75; adhesive removal test: SMD = -2.60, 95% CI: -4.27, -0.93; morris water maze test: SMD = -3.91, 95% CI: -7.03, -0.79), and reduced the release of proinflammatory factors (IL-1β: SMD = -2.02, 95% CI: -2.77, -1.27; IL-6: SMD = -3.01, 95% CI: -4.47, -1.55; TNF-α: SMD = -2.72, 95% CI: -4.30, -1.13), increasing the number of neurons (apoptosis rate: SMD = -2.24, 95% CI: -3.32, -1.16; the number of neurons: SMD = 3.70, 95% CI: 2.44, 4.96). The funnel plots for the two main outcome measures were asymmetric, indicating publication bias. The median score on the CAMARADES checklist was 7 points (IQR: 6–9).

**Conclusions:**

This meta-analysis shows that EEVs are superior to natural EVs for the treatment of IS. However, research in this field is still at an early stage, and more research is needed to fully understand the potential therapeutic mechanism of EEVs and their potential use in the treatment of IS.

**PROSPERO registration number:**

CRD42022368744.

**Supplementary Information:**

The online version contains supplementary material available at 10.1186/s12951-023-02114-8.

## Introduction

Ischemic stroke (IS) is a common neurological disease and a leading cause of disability and death [1, 2]. Despite significant advances in the treatment of IS over the past decade, current treatment options are still limited. Tissue plasminogen activator is the only drug approved by the Food and Drug Administration (FDA) for IS treatment, which has a very narrow treatment window of 4.5 h [3, 4]. Mechanical thrombectomy is also only available to 10% of patients [5]. Therefore, there is an urgent need to develop new therapeutic strategies to improve IS prognosis.

Natural extracellular vesicles (EVs) are small vesicles released by cells, carrying a variety of bioactive molecules, such as proteins, lipids and nucleic acids [6]. EVs include exosomes, microvesicles (MVs) and apoptotic bodies [7]. Existing preclinical studies and clinical trials have demonstrated the potential of EVs to treat IS [8]. The biological advantages of EVs include low immunogenicity, natural stability and high delivery efficiency. In addition, EVs can easily cross the blood-brain barrier (BBB), indicating their availability to target central nervous system diseases [9, 10]. Research shows that EVs can regulate angiogenesis, inhibit inflammation, inhibit neuronal apoptosis, reduce cell remodeling, and improve the microenvironment of post-IS brain injury via delivering bioactive molecules to the brain [11–15]. For example, stem cell-derived EVs have been shown to up-regulate the expression of genes of multiple angiogenesis pathways such as vascular endothelial growth factor (VEGF), angiopoietin 1 (ANG1), angiopoietin 2 (ANG2), matrix metallopeptidase 9 (MMP9), thrombopoietin 1 (TSP1), so as to promote the formation of new blood vessels and improve cerebral blood flow [16]. EVs can also deliver brain-derived neurotrophic factor (BDNF) to the brain and promote the survival and growth of neurons [17].

However, EVs limitations suggest low yield, low biological activity, weak targeting and rapid clearance in vivo [8]. To address the limitations of natural EVs as a treatment option for IS, researchers are developing engineered extracellular vesicles (EEVs) through bioengineering techniques [12, 13]. EEVs can be produced in two ways: by engineering modification on EV-producing parent cells or by directly modifying EVs [12]. The first method involves co-incubating the parent cells with targeted molecules or transfecting plasmid into the parent cells to create EVs with specific targeted molecules [18]. The second method involves direct modification of EVs using techniques such as incubation, ultrasonic treatment, electroporation, antibody-specific loading, freeze-thaw methods, and saponin-assisted method [18].

Although there have been many preclinical studies on the treatment of IS with EEVs, there is still a lack of evidence-based research. Therefore, this systematic review and meta-analysis aimed to compare the efficacy differences between EEVs and natural EVs in preclinical studies, providing a basis for future research in this field.

## Methods

This systematic review was conducted in accordance with the Preferred Reporting Items for Systematic Reviews and Meta-Analyses (PRISMA) guidelines [19], and the protocol for this meta-analysis has been published in the PROSPERO database with the registration number CRD42022368744.

### Search strategy

We conducted a comprehensive literature review to identify relevant studies published in PubMed, EMBASE, Web of Science, Cochrane Library, and Scopus databases until August 1, 2023. We also manually searched for references in relevant reviews and meta-analyses. We aimed to capture all relevant studies on the therapeutic use of EEVs for IS. Our search strategy, therefore, included the use of MeSH terms and free words “extracellular vesicles” and “stroke,“ and we did not impose any restrictions on the term “engineered.“ Details of our retrieval strategies for each database can be found in the supplementary materials.

### Study selection

After removing duplicates, we screened titles and abstracts to exclude clearly irrelevant studies and then read the full text of the remaining studies to screen them further based on inclusion and exclusion criteria. The inclusion criteria included: (a) studies published in English, (b) studies conducted on IS animal models, (c) studies providing detailed methods for extracting, engineering, and identifying EVs, (d) Studies that measure at least one of the following: infarct volume or neurological scores. The exclusion criteria included: (a) studies with data that could not be extracted, (b) studies that do not compare the treatment effects of EEVs versus natural EVs, (c) non-rodent studies. Two authors (RY, SHW) independently performed the study selection, and any discrepancies were resolved through discussion with a third author (QH).

### Data extraction

We extracted the following information from the included studies: (a) General Information: First author, year of publication, and country. (b) Animal Characteristics: Species, age, gender, weight, and number. (c) EVs Characteristics: Source, extraction method, diameter, engineered method, and engineering targets. (d) Details of EEVs Treatment: Route, dosage, and time. (e) Duration of Follow-Up. (f) Outcome Measures: Infarct Volume: Percentage of the infarct volume and the size of the infarct volume. Neurological Scores: Modified neurological severity score (mNSS) and Zea-Longa score. Behavioral Recovery: Rotarod test, grid-walking test, adhesive removal test, and Morris water maze test. Pro-inflammatory Factor Release: IL-1β, IL-6, and TNF-α. Cellular Effects: Apoptosis rate and neuron numbers .

For studies with only graphical data available, we used the online tool WebPlotDigitizer (https://automeris.io/WebPlotDigitizer/) to extract data from the graphs. If multiple time points were involved throughout the measurements, we extracted data only from the latest time point. Two authors (YHC, JBC) independently performed data extraction, and any differences of less than 10% were averaged while any differences greater than 10% were discussed and resolved with a third author (LY). If additional information was needed, we contacted the authors via email. We excluded these studies if data were still unavailable after two attempts.

### Risk of bias assessment

We assessed the quality of the studies using the Collaborative Approach to Meta-analysis and Review of Animal Data in Experimental Studies (CAMARADES) bias risk checklist [20]. This checklist comprises: (a) publication in a peer-reviewed journal, (b) statement of temperature control, (c) randomization, (d) allocation concealment, (e) blinded outcome assessment, (f) avoidance of clearly biased anesthetics, (g) use of appropriate animal models, (h) sample size calculation, (i) compliance with animal welfare regulations, (j) statement of potential conflicts of interest. Two independent authors (XYL and XZ) conducted a risk assessment and any discrepancies were resolved through discussion with a third author (RY).

### Statistical analysis

We used Revman 5.4 software for data analysis. Continuous variables were presented as standardized mean difference (SMD) with 95% confidence intervals (CI). We tested for heterogeneity in each outcome measure using the Q-test and I^2^ statistic. Based on the heterogeneity, we used a fixed-effects model only when no significant heterogeneity was observed (*p* > 0.1, I^2^ < 50%). Otherwise, a random-effects model was employed for meta-analysis, with *p* < 0.05 considered statistically significant. When significant heterogeneity was present, we conducted sensitivity analysis by excluding individual studies one by one and performed subgroup analysis to determine the source of heterogeneity.

## Results

### Study characteristics

**Fig. 1:**
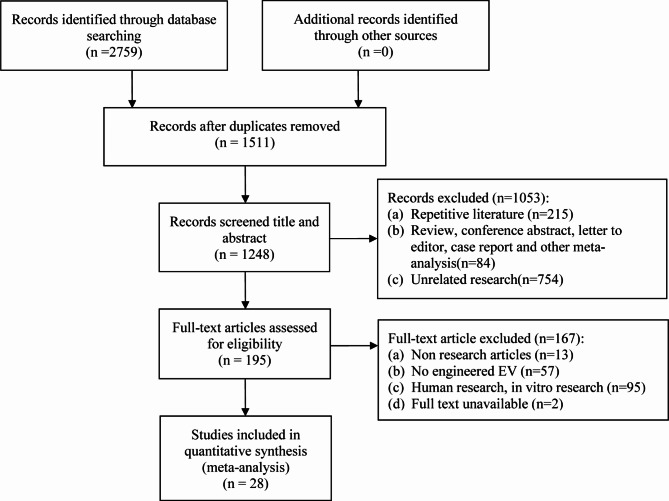
PRISMA flow diagram. Summary of the number of studies identified, screened, and ultimately included in the systematic review and meta-analysis

We identified 2793 studies from the databases, which we then screened based on our inclusion and exclusion criteria. As shown in Fig. 1 and 28 studies [17, 21–47] ultimately met our criteria and were included in this review. Details of these studies are presented in Table 1. All studies were conducted using rats (n = 19) and mice (n = 9). Apart from two studies that utilized photochemistry and electrocoagulation techniques, the prevalent approach was the suture method of middle cerebral artery occlusion (MCAO) (n = 26). Mesenchymal stem cells (MSC) were the primary source of EVs in most studies (n = 15), with other sources including neural stem cells (NSC) (n = 5), blood (n = 5), and soma (n = 3). The predominant method of engineering EVs was through lentiviral transfection (n = 16), followed by coculture (n = 7), ultrasonic techniques (n = 3), electroporation (n = 1), and surface modification (n = 1). The preferred route of EEVs administration was intravenous injection (n = 21), though some studies opted for intracerebral injection (n = 5) or nasal administration (n = 2). Administration timing varied, spanning from a day before IS (n = 2) to 14 days post-IS (n = 26), with select studies administering EVs on multiple occasions (n = 5). Notably, a significant portion of studies engineered the parent cells (n = 19), as opposed to directly engineering the EVs (n = 9).

**Table 1:** Summary of studies included in the systematic review

Study	Specie/gender/age/weight	Number	Stroke model	Characteristics of EVs				Therapeutic method			Duration
				Source	Diameter(nm)	Methods of engineered	Engineering targets	Route	Dose	Time	
Deng et al., 2019 [21]	C57BL/6J mice, 8-10w, M	40	Suture	MSC	NA	Transfection	Cell	IV	NA	24 h-14d	14d
Guo_1 et al., 2020 [22]	SD rat, M, 250-300 g	39	Suture	Blood	150–165	Ultrasonic	EV	IV	10 mg/kg	24 h-7d	28d
Guo_2 et al., 2021 [23]	SD rat, M, 250-300 g	36	Suture	Blood	NA	Surface modification	EV	IV	NA	Immediately	24 h
Huang et al., 2020 [24]	SD rat, M	84	Suture	Blood	81.8-133.9	Ultrasonic	EV	IV	1.6 mg	48 h	1d
Jiang et al., 2022 [25]	T2DM	28	Electrocoagulation	NSC	146 ± 17	Coculture	EV	Transcranial	10μL	2 h	21d
Kim_1 et al., 2019 [26]	SD rat, 10w, M, 280-320 g	30	Suture	Soma	143.5 ± 5.7	Electroporation	EV	IV	30 μg	Before 18 h	24 h
Kim_2 et al., 2020 [27]	SD rat, 9w, M, 250-300 g	34	Suture	MSC	194.2 ± 44.5	Coculture	Cell	IV	200 μg	Immediately	28d
Kim_3 et al., 2021 [28]	SD rat, 10w, M, 280-320 g	56	Suture	Soma	39.1 ± 5.1	Transfection	Cell	Nasal	75 μg	2 h	3d
Lee et al., 2016 [29]	SD rat, M, 200-250 g	42	Suture	MSC	NA	Coculture	Cell	IV	0.2 mg/kg	48 h	7d
Li et al., 2020 [30]	SD rat, M, 250-300 g	135	Suture	Blood	68.06 ± 1.94	Coculture	EV	IV	3 mg/kg	24 h-7d	7d
Liu et al., 2022 [31]	SD rat, M, 250-280 g	45	Suture	Blood	NA	Ultrasonic	EV	IV	0.75 mg	Immediately	24 h
Pan et al., 2020 [32]	C57BL/6J mice, 6-8w, M	40	Suture	MSC	24.8-169.3	Transfection	Cell	IV	1 × 10^10^	1.5 h	4d
Shi et al., 2022 [33]	SD rat, 8-12w, M, 250-300 g	84	Suture	MSC	99.2–388.0	Coculture	EV	Transcranial	100 lg/kg	24 h-3d	28d
Wang et al., 2020 [34]	T2DM, 8-10w, M, 42-48 g	143	Suture	MSC	129.4 ± 19.9	Transfection	Cell	IV	50 μg	2 h	14d
Wei et al., 2022 [35]	SD rat, M, 280-320 g	24	Suture	MSC	30–100	Transfection	Cell	Transcranial	5μL	Immediately	21d
Xin_1 et al., 2017 [36]	Wistar rat, 2-3 m, M, 270-300 g	24	Suture	MSC	NA	Transfection	Cell	IV	100 μg	24 h	28d
Xin_2 et al., 2021 [37]	Wistar rat, 2-3 m, M, 250-300 g	30	Suture	MSC	NA	Transfection	Cell	IV	100 μg	24 h	28d
Xu_1 et al., 2020 [38]	C57BL/6J mice, M, 25 ± 2 g	32	Suture	MSC	30–100	Transfection	Cell	IV	400 μg	Immediately	7d
Xu_2 et al., 2022 [17]	C57BL/6J mice, 9.2 ± 0.4w, M, 23 ± 1.2 g	24	Suture	NSC	100–150	Transfection	Cell	IV	100uL	2 h	24 h
Yang_1 et al., 2020 [39]	C57BL/6J mice, 8-10w, M, 24-26 g	430	photochemistry	Soma	117.3 ± 4.8	Transfection	Cell	IV	12 mg/kg	24 h	28d
Yang_2 et al., 2020 [40]	SD rat, M, 280-350 g	118	Suture	MSC	30–210	Transfection	Cell	IV	100 μg	24 h	14d
Yang_3 et al., 2022 [41]	C57BL/6J mice, M, 20-25 g	24	Suture	MSC	28–211	Transfection	Cell	IV	10 μg	Before 24 h	2 h
Yoon et al., 2022 [42]	SD rat, 8w, M, 300-330 g	60	Suture	NSC	39.9-339.8	Transfection	Cell	IV	300 μg/ kg	2 h	24 h
Zhang et al., 2020 [43]	SD rat, 8w, M, 240-280 g	38	Suture	NSC	126.1 ± 7.2	Coculture	Cell	Transcranial	4 × 10^9^	24 h	14d
Zhao et al., 2020 [44]	SD rat, M, 260-280 g	24	Suture	MSC	30–150	Transfection	Cell	IV	200μL	24 h and 14d	28d
Zhou_1 et al., 2022 [45]	SD rat, 6-8w, M, 200-240 g	NA	Suture	MSC	30–200	Transfection	Cell	IV	NA	2 h	28d
Zhou_2 et al., 2023 [46]	C57BL/6J qmice, 9-10w, M, 23-27 g	36	Suture	MSC	100	Transfection	Cell	Nasal	10uL	2 h	28d
Zhu et al., 2022 [47]	SD rat, 8w, M, 250-280 g	60	Suture	NSC	30–200	Coculture	EV	Transcranial	10μL	72 h	28d

SD rat: Sprague-Dawley rat; T2DM: type 2 diabetes mellitus mice; M: Male; BMSC: Bone marrow mesenchymal stem cell; NSC: Neural stem cell; IV:Intravenous injection; NA: Not Applicable

### Outcomes

#### EEVs reduce infarct volume and improve neurological scores after IS

**Fig. 2:**
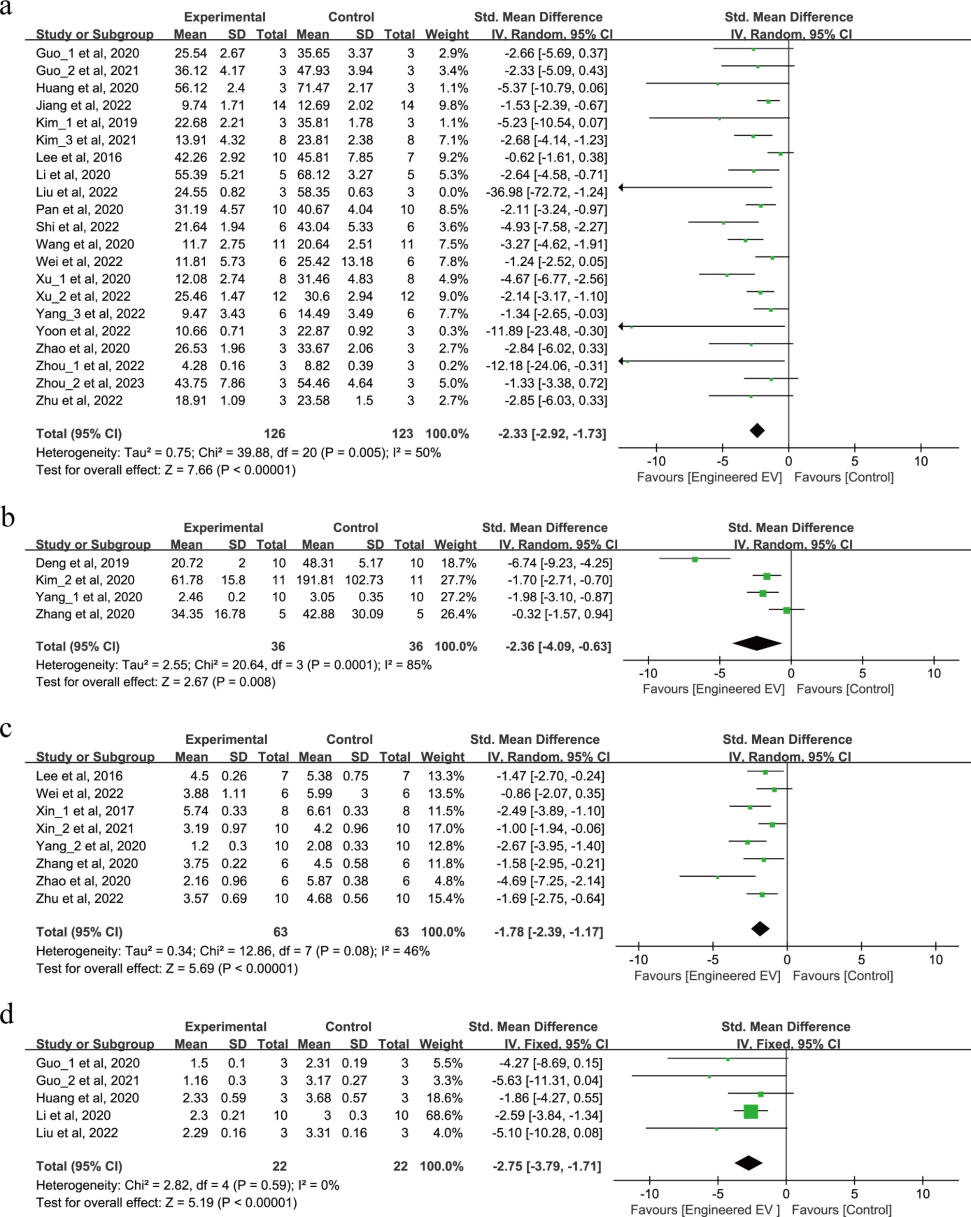
Forest plots show the effect of EEVs therapy on infarct volume and neurological scores in IS. (**a**) The percentage of infarct volume. (**b**) The size of infarct volume. (**c**) MNSS. (**d**) Zea-Longa score

The effects of EEVs therapy on infarct volume and neurological scores were shown in Fig. 2a-d. A total of 321 animals in 25 studies reported changes in infarct volume after treatment with EEVs, of which 21 studies reported the percentage of infarct volume (Fig. 2a) and 4 studies reported the size of infarct volume (Fig. 2b). The results showed that the EEVs reduced the percentage of infarct volume (SMD = -2.33, 95% CI: -2.92, -1.73, *p* < 0.00001, Tau^2^ = 0.75, I^2^ = 50%) and the size of infarct volume (SMD = -2.36, 95% CI: -4.09, -0.63, *p* = 0.008, Tau^2^ = 2.55, I^2^ = 85%) compared to natural EVs therapy.

Furthermore, we examined the effect of EEVs therapy on neurological scores after IS. In 8 studies, 126 animals were assessed using the modified neurological severity score (mNSS) (Fig. 2c), and 44 animals in 5 studies used the Zea-Longa score (Fig. 2d). The results showed that treatment with EEVs significantly improved mNSS after IS (SMD = -1.78, 95% CI: -2.39, -1.17, *p* < 0.00001, Tau^2^ = 0.34, I^2^ = 46%). Similarly, the Zea-Longa score demonstrated comparable results (SMD = -2.75, 95% CI: -3.79, -1.71, *p* < 0.00001, I^2^ = 0%).

#### EEVs promote behavioral recovery after IS

**Fig. 3:**
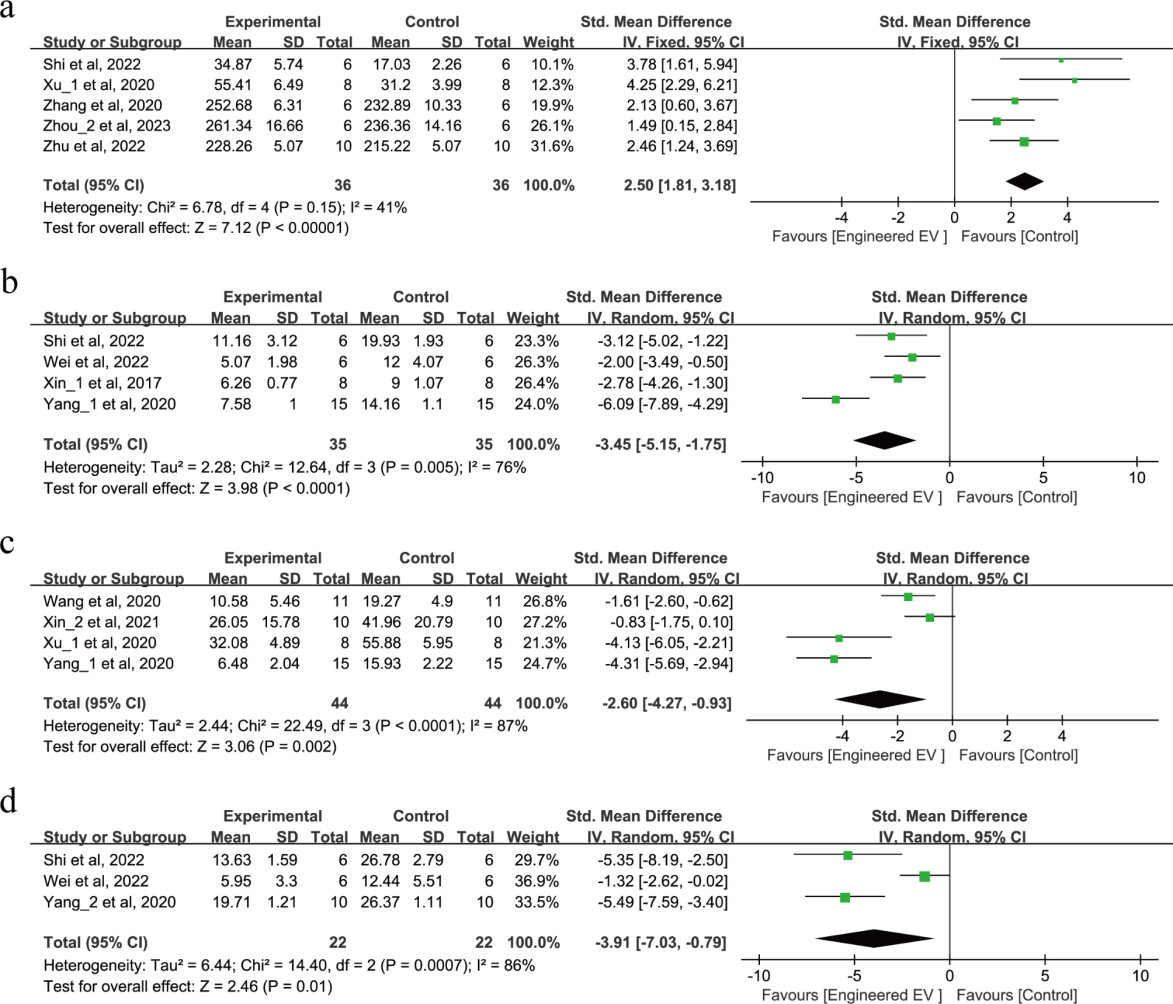
The forest plot of the effect of EEVs treatment on IS behavior is shown. (**a**) Rotarod test. (**b**) Grid-walking test. (**c**) Adhesive removal test. (**d**) Morris water maze test

Behavioral tests were conducted on a total of 274 animals across 11 studies as shown in Fig. 3a-d. For motor and coordination function, 5 studies performed the rotarod test (SMD = 2.50, 95% CI: 1.81, 3.18, *p* < 0.00001, I^2^ = 41%) as shown in Fig. 3a, while 4 studies performed the grid-walking test (SMD = -3.45, 95% CI: -5.15, -1.75, *p* < 0.0001, Tau^2^ = 2.28, I^2^ = 76%) as shown in Fig. 3b. For motor and sensory function, 4 studies performed adhesive removal test (SMD = -2.60, 95% CI: -4.27, -0.93, *p =* 0.002, Tau^2^ = 2.44, I^2^ = 87%) as shown in Fig. 3c. For learning and memory function, 3 studies performed the morris water maze test (SMD = -3.91, 95% CI: -7.03, -0.79, *p* = 0.01, Tau^2^ = 6.44, I^2^ = 86%) as shown in Fig. 3d. In summary, all these tests suggest that treatment with EEVs improves behavioral recovery after IS.

#### EEVs reduce the release of pro-inflammatory factors after IS

**Fig. 4:**
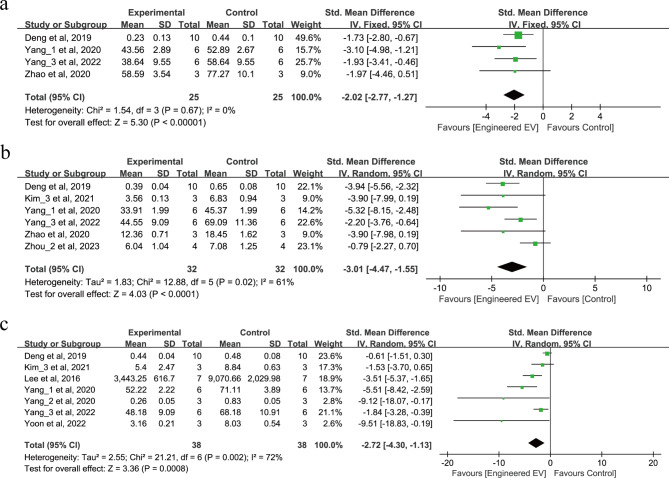
Forest plot of the effect of EEVs treatment on pro-inflammatory factor release after IS. (**a**) IL-1β. (**b**) IL-6. (**c**) TNF-α.

9 studies involving 190 animals reported the release of pro-inflammatory factors after IS as shown in Fig. 4a-c. 4 studies reported that EEVs can reduce IL-1β (SMD = -2.02, 95% CI: -2.77, -1.27, *p* < 0.00001, I^2^ = 0%) as shown in Fig. 4a. 6 studies reported that EEVs can reduce the release of IL-6 (SMD = -3.01, 95% CI: -4.47, -1.55, *p* < 0.0001, Tau^2^ = 1.83, I^2^ = 61%) as shown in Fig. 4b. 7 studies reported that EEVs can also reduce the release of TNF-α (SMD = -2.72, 95% CI: -4.30, -1.13, *p* = 0.0008, Tau^2^ = 2.55, I^2^ = 72%) as shown in Fig. 4c. In summary, these studies all demonstrate that treatment with EEVs can reduce the release of pro-inflammatory factors after IS.

#### EEVs reduce apoptosis rate and increase the number of neurons after IS

**Fig. 5:**
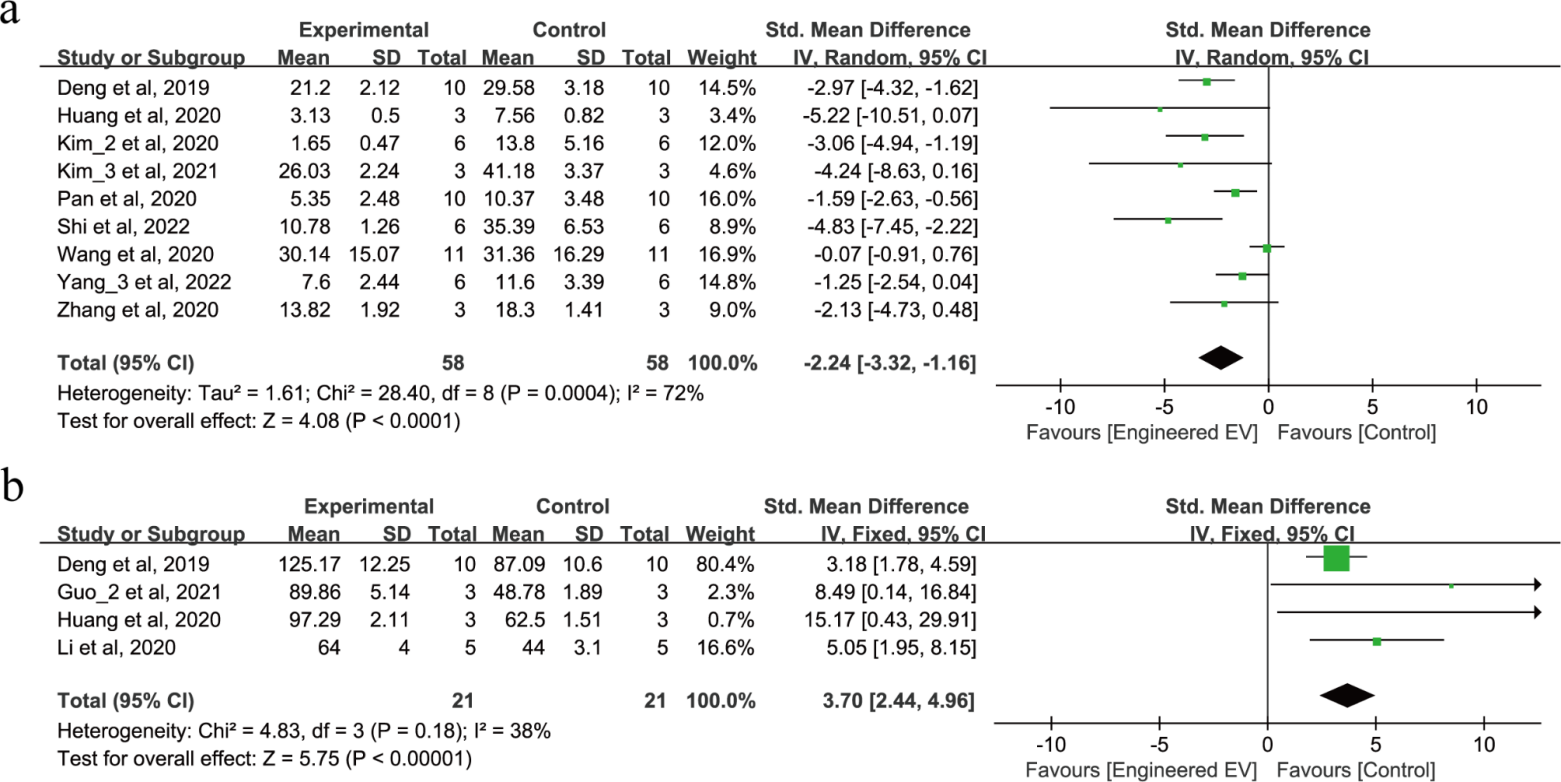
Forest plot of the effect of EEVs treatment on apoptotic rate and the number of neurons after IS. (**a**) Apoptotic rate. (**b**) The number of neurons

11 studies involving 158 animals reported on the apoptosis rate and the number of neurons after IS, as shown in Fig. 5a-b. 9 studies reported that treatment with EEVs reduce apoptosis rate (SMD = -2.24, 95% CI: -3.32, -1.16, *p* < 0.0001, Tau^2^ = 1.61, I^2^ = 72%) as shown in Fig. 5a. 4 studies reported that treatment with EEVs significantly increase neuron numbers after IS (SMD = 3.70, 95% CI: 2.44, 4.96, *p* < 0.00001, I^2^ = 38%) as shown in Fig. 5b.

### Subgroup and sensitivity analyses

We conducted a subgroup analysis to explore the source of heterogeneity. As shown in Table 2, we did not observe significant sources of heterogeneity in the outcome of infarct volume among subgroups of randomization, blinding, animal species, source of EVs, methods of engineering, engineering targets, route of administration, and the timepoint of administration. We also conducted a sensitivity analysis to ensure the robustness of determining the overall effect size of the observed outcome measurements. We removed one study at a time and recalculated the pooled effect size for the remaining studies. The results showed that for all outcomes, there was no significant improvement in heterogeneity between studies, indicating that no study had driven the source of heterogeneity.

**Table 2:** Subgroup analysis of infarct volume

Factor	Number of study	Number of animal	SMD (95% CI)	I^2^ (%)	Q Statistic *(P-*value)	Subgroup analysis *P*-value
Randomisation						0.28
Yes	13	165	-2.13(-2.81, -1.45)	49	23.63(0.02)	
No	8	84	-2.94(-4.26, -1.62)	53	14.81(0.04)	
Blinding						0.26
Yes	7	156	-1.95(-2.82, -1.08)	51	12.21(0.06)	
No	14	116	-2.65(-3.49, -1.80)	51	26.29(0.02)	
Animal species						0.47
SD rat	14	121	-2.66(-3.71, -1.62)	53	27.60(0.01)	
C57BL/6J mice	5	78	-2.16(-3.04, -1.29)	47	7.57(0.11)	
Source of EVs						0.78
MSC	10	137	-2.36(-3.27, -1.45)	66	26.50(< 0.01)	
NSC	4	64	-1.94(-2.84, -1.04)	27	4.08(0.25)	
Blood	5	34	-2.83(-4.36, -1.31)	11	4.52(0.34)	
Soma	2	28	-2.47(-5.33, 0.39)	39	1.65(0.20)	
Methods of engineered						0.66
Transfection	12	146	-2.34(-3.05, -1.64)	45	18.22(0.05)	
Coculture	5	73	-2.05(-3.24, -0.85)	65	11.42(0.02)	
Ultrasonic	3	18	-4.49(-9.79, 0.81)	51	4.12(0.13)	
Engineering targets						0.20
Cell	10	123	-1.92(-2.71, -1.13)	53	19.28(0.02)	
EVs	9	86	-2.85(-4.02, -1.67)	39	13.04(0.11)	
Route of administration						0.54
IV	15	177	-2.57(-3.73, -1.78)	53	29.62(< 0.01)	
Transcranial	4	58	-2.11(-3.37, -0.86)	56	6.81(0.36)	
Timepoint of administration						0.58
Pretreatment	2	26	-2.53(-4.92, -0.14)	28	1.38(0.24)	
0-24 h	12	174	-2.20(-2.89, -1.52)	51	22.23(0.02)	
25-72 h	3	29	-2.00(-4.43, 0.43)	54	4.34(0.11)	
Multiple	4	34	-3.20(-4.48, -1.93)	0	2.11(0.55)	

SD rat: Sprague-Dawley rat; IV:Intravenous injection; CI: Confidence interval; SMD: standardized mean difference

### Research quality and bias risk

As shown in Table 3, the median quality assessment score for the studies was 7 points (IQR: 6–9). However, most studies employed the principle of random allocation and only a few reported concealment of allocation. Half of the studies used a blinding to evaluate the results. Only one study provided information on sample size calculation, which received a risk of bias score of 10 points, as shown in Table 4.

**Table 3:** CAMARADES Checklist Assessment Bias Risk

Checklist Item	Number of study	Percentage
1. Peer reviewed	28	100.0
2.Temperature control description	25	89.3
3. Random allocation to group	17	60.7
4. Allocation concealment	11	39.3
5. Blinded assessment outcome	12	42.9
6. Appropriate animal models	28	100.0
7. Suitable for anesthetics	25	89.3
8. Sample size calculation	1	3.6
9. Animal welfare regulations	28	100.0
10. Conflict of interest	25	89.3
Median study quality (IQR)		7 (6–9)

**Table 4:** Extended risk of bias checklist data

Study	Peer reviewed	Temperature control	Random allocation	Allocation concealment	Blinded assessment	Appropriate animal models	Suitable for anesthetics	Sample size calculation	Animal welfare regulations	Conflict of interest	Total Score
Deng et al., 2019	√	√	√	√		√	√		√	√	8
Guo_1 et al., 2020	√		√	√		√			√	√	6
Guo_2 et al., 2021	√	√	√			√			√	√	6
Huang et al., 2020	√	√				√	√		√	√	6
Jiang et al., 2022	√	√	√			√	√		√	√	7
Kim_1 et al., 2019	√					√	√		√		4
Kim_2 et al., 2020	√	√				√	√		√	√	6
Kim_3 et al., 2021	√	√			√	√	√		√		6
Lee et al., 2016	√	√	√	√	√	√	√		√	√	9
Li et al., 2020	√	√	√			√	√		√		6
Liu et al., 2022	√					√			√	√	4
Pan et al., 2020	√	√				√	√		√	√	6
Shi et al., 2022	√	√			√	√	√		√	√	7
Wang et al., 2020	√	√	√	√	√	√	√		√	√	9
Wei et al., 2022	√	√	√	√		√	√		√	√	8
Xin_1 et al., 2017	√	√			√	√	√		√	√	7
Xin_2 et al., 2021	√	√			√	√	√		√	√	7
Xu_1 et al., 2020	√	√	√	√	√	√	√		√	√	9
Xu_2 et al., 2022	√	√	√	√	√	√	√		√	√	9
Yang_1 et al., 2020	√	√	√	√	√	√	√	√	√	√	10
Yang_2 et al., 2020	√	√	√	√	√	√	√		√	√	9
Yang_3 et al., 2022	√	√				√	√		√	√	6
Yoon et al., 2022	√	√				√	√		√	√	6
Zhang et al., 2020	√	√	√	√	√	√	√		√	√	9
Zhao et al., 2020	√	√	√	√	√	√	√		√	√	9
Zhou_1 et al., 2022	√	√	√			√	√		√	√	7
Zhou_2 et al., 2023	√	√	√			√	√		√	√	7
Zhu et al., 2022	√	√	√			√	√		√	√	7

### Publication bias

**Fig. 6:**
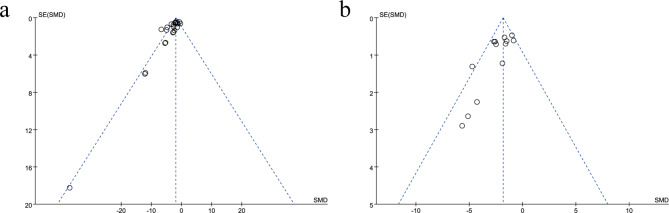
Publication bias funnel plots for infarct volume and neurological scores. (**a**) Infarct volume. (**b**) Neurological scores

We also conducted a publication bias test and generated funnel plots for outcome measures that included more than ten studies. The results indicated publication bias for both of our outcome measures. The funnel plots for infarct volume and neurological scores appeared asymmetrical, as illustrated in Fig. 6, with a majority of the studies indicating more positive effects of EEVs.

## Discussion

**Fig. 7:**
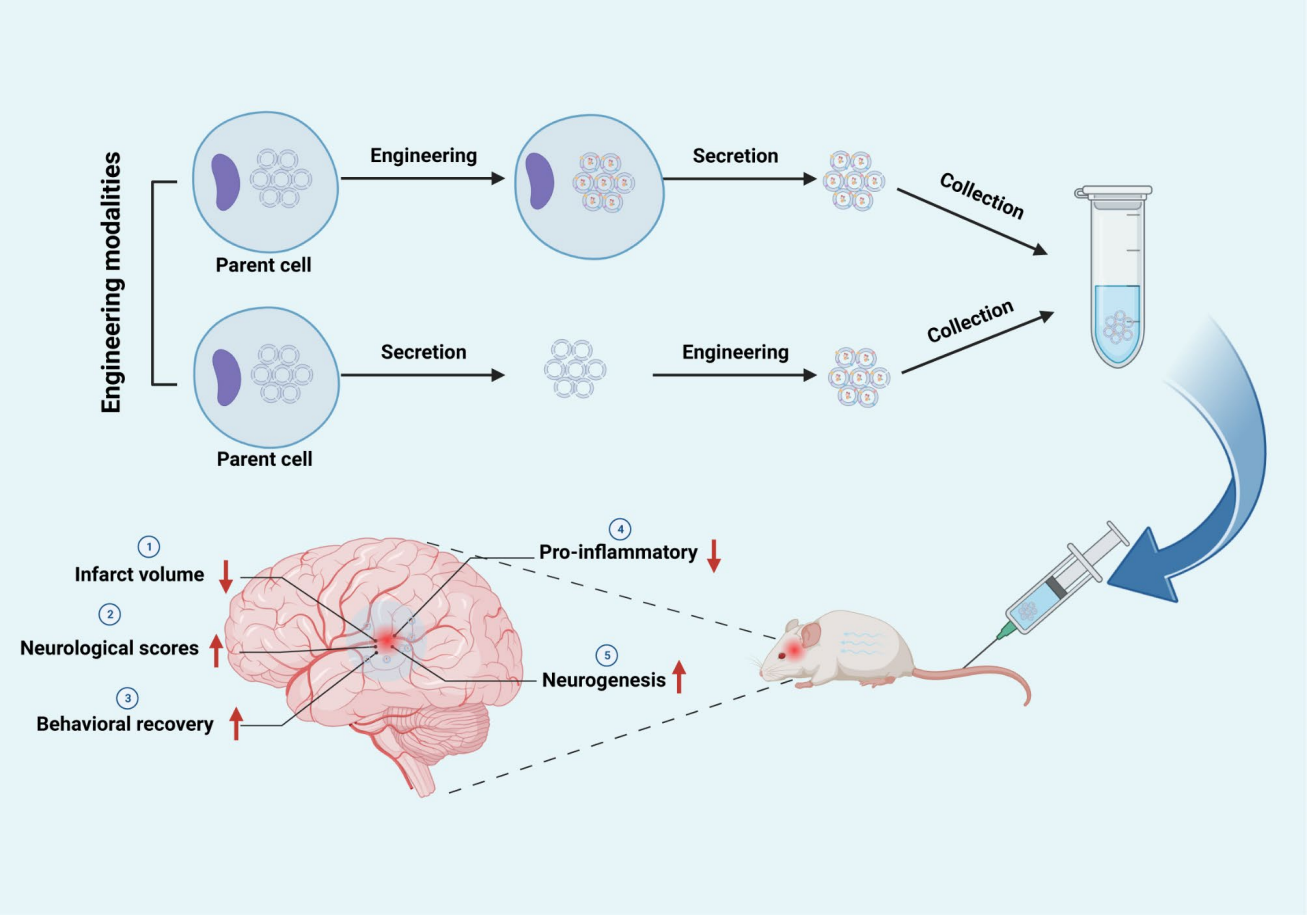
Two engineering modalities of EVs and their therapeutic effects on ischemic stroke

Our meta-analysis of 28 published preclinical studies examined the therapeutic effects of EEVs on IS. Our findings showed that treatment with EEVs significantly reduced infarct volume, improved neurological function, and promoted behavioral recovery compared to treatment with native EVs. The observed benefits of EEVs may be attributed to their ability to inhibit apoptosis, increase the number of neurons, and reduce the release of pro-inflammatory factors, as shown in Fig. 7.

### Research bias and heterogeneity

As with any meta-analysis, it is essential to consider the potential risks of bias and heterogeneity in the included studies. We assessed the risk of bias using the CAMARADES checklist and found that most studies had a low risk of bias. The median quality assessment score was 7 (IQR: 6–9). As most trials employed random grouping, we did not observe detailed calculations for allocation concealment and sample size. Regarding heterogeneity, we observed moderate to high levels of heterogeneity across the studies, which may be due to differences in sources of EVs, engineered methods, and treatment methods. Despite these limitations, the consistent direction and magnitude of the effect across the studies support the overall conclusions of our meta-analysis.

### The potential therapeutic mechanism of EEVs

The potential therapeutic mechanism of EEVs in the treatment of IS is thought to be similar to that of natural EVs. Studies suggest that EVs improve the immune microenvironment at IS sites, inhibit inflammatory reactions, counter cell apoptosis, and promote vascular and nerve remodeling [48–50]. However, EEVs may have greater therapeutic potential due to their ability to be engineered to target specific cells or pathways. For example, a study by Yang et al. [40] found that EEVs loaded with circular RNA SCMH1 (circSCMH1) were able to reduce the inhibition of methylCpG-binding protein 2 (MeCP2) target gene transcription, leading to enhanced neuronal plasticity, inhibition of glial cell activation, and improved functional recovery in rats and monkeys after stroke. Another study by Deng et al. [21] found that upregulation of miR-138-5p in EEVs derived from bone marrow mesenchymal stem cells successfully targeted LCN2, promoting astrocyte proliferation, inhibiting inflammatory reactions, inhibiting cell apoptosis, and reducing nerve injury. Similarly, Pan et al. [32] found that EVs rich in miR-132-3p may reduce the production of reactive oxygen species (ROS), BBB dysfunction and brain injury in vascular endothelial cells injured by hypoxia/reperfusion (H/R) by activating PI3K and eNOS. Although EEVs appear promising in targeting specific cellular functions, the research in this domain remains limited, further research is necessary to comprehensively grasp the therapeutic potential of EEVs in IS treatment. Before EEVs can be extensively adopted for IS treatment, conducting additional clinical trials to ascertain their safety and efficacy is imperative.

### Preparation of EEVs

There are two strategies for preparing EEVs [8]. The first involves engineering the parent cells, such as pre-treating or transfecting with specific molecules. The second strategy is to directly engineer the EVs with precision, using techniques like electroporation, co-incubation, antibody-specific loading, heat shock or freeze-thaw methods, and ultrasonic treatment. These strategies have been successfully implemented in the field of EEVs. The subgroup analysis in our study explored the differences in the effects of these two strategies on reducing infarct volume. Approximately half of the studies involved engineering the parent cells, and our observations suggest that directly engineering the EVs might be more effective in reducing infarct volume compared to engineering the parent cells. However, this difference was not statistically significant and further research is needed for validation. Utilizing parent cells to produce EEVs offers certain advantages: it is simpler and more convenient, and it retains the biophysical characteristics and stability of the EVs. However, this method also carries inherent risks. Overexpression of certain molecules in cells can trigger complex biological reactions, possibly compromising the bioactivity of EVs [8]. In contrast, direct engineering of EVs may provide greater control, ensuring precision in loading, targeting, and delivering the functions of EVs. Current engineering modifications to EVs mainly focus on enhancing their loading capacity, circulation time, and targeting abilities to achieve better therapeutic outcomes.

### Loading capacity of EEVs

The ability to deliver various substances to specific cells makes EVs promising candidates for drug delivery. Research indicates that enhancing the miRNA content in EVs can potentially ameliorate ischemic brain injury [34, 37, 47]. For example, Wang et al. [34] found that EEVs rich in miR-126 are more effective than natural EVs in treating diabetes-induced ischemia by reducing acute injury and promoting neural recovery. Similarly, Xin et al. [37] showed that secretion rich in miR-17-92 increased axonal elongation and myelin formation in rats by down-regulating the PTEN-induced PI3K/Akt/mTOR pathway, thus aiding in nerve function recovery post-middle cerebral artery occlusion. In addition, EVs have been explored as drug delivery vehicles. Engineering technologies can facilitate the loading of commonly used drugs for treating ischemic injuries into EVs. Such approaches can reduce the damage and inactivation of drugs during transportation, improving their bioavailability and specificity. For example, Zhu et al. [47] showed that EEVs loaded with brain-derived neurotrophic factor (BDNF) not only inhibited the activation of microglia after stroke but also promoted the differentiation of endogenous neural stem cells into neurons. Guo et al. [23] found that EEVs loaded with quercetin (Que) can activate Nrf2/HO-1 pathway to inhibit ROS production and improve the survival rate of neurons.

### Circulation time and targeting of EVs

In recent years, researchers have increasingly focused on enhancing the stability, circulation half-life, and targeting capabilities of EVs within the body. Although previous studies have shown that EVs are often rapidly cleared or concentrated in the liver, spleen, and lungs after injection into animal models, the therapeutic effect of EVs is closely related to their half-life and effective concentration in the lesion area [51, 52]. To extend the circulation half-life, scientists have begun to use nanotechnology to encapsulate EVs, thereby reducing their non-specific interactions with other cells, and subsequently enhancing the stability and half-life of EVs [53, 54]. For instance, Liu et al. [54] used hyaluronic acid hydrogel to encapsulate exosomes derived from bone marrow mesenchymal stem cells, thus achieving higher stability and promoting brain structure reconstruction and neurological function recovery. Additionally, to enhance the targeting capabilities of EVs, researchers have started to engineer the EVs. For example, Tian et al. [55] coupled c(RGDyK) peptide to the surface of EVs, enabling the intravenously injected cRGD-Exo to specifically target ischemic brain lesion areas. Alvarez et al. [56] enhanced targeting to neurons by engineering dendritic cells to express Lamp2b (an EV membrane protein) fused with neuron-specific RVG peptide. Kim et al. [27] also developed magnetic EVs fused with iron oxide nanoparticle (IONP) as a bioengineering means to enhance the targeting ability of EVs. In summary, through nanotechnology and engineering approaches, researchers are actively exploring ways to prolong the circulation half-life and enhance the targeting capability of EVs to optimize their therapeutic applications.

### Limitations

Our review has some limitations. First, only a few studies were included. Significant differences exist in the following aspects of the covered studies: source, injection route, treatment time, dose, and follow-up time of EVs in the covered studies. Additionally, there are concerns about data deviation and accuracy which require further evaluation. Future research should be more comprehensive to address these limitations.

## Conclusions

Our study highlights the advantages of EEVs in the treatment of IS. Compared to natural EVs, they have shown a stronger therapeutic effect, especially in reducing infarct volume, enhancing neural function, promoting behavioral recovery, reducing inflammatory responses, regulating cell apoptosis, and increasing the number of neurons. These findings open a new perspective for the in-depth study of EV engineering techniques. However, the production and optimization of EEVs do face challenges in terms of time and cost. Although this field remains in its exploratory phase, further investigations are imperative to deepen our grasp of the therapeutic potential of EEVs, particularly in the context of IS treatment. Before the clinical introduction of EEVs, it is crucial to conduct further clinical trials to ensure both their safety and efficacy.

### Electronic supplementary material

Below is the link to the electronic supplementary material.

Supplementary Material 1

## Data Availability

All the data that underlie the conclusions in this research are available online.

## Abbreviations

ADSC: Adipose-derived stromal cell
ANG1: Angiopoietin 1
ANG2: Angiopoietin 2
BBB: Blood-brain barrier
BDNF: Brain-derived neurotrophic factor
BMSC: Bone marrow mesenchymal stem cell
CAMARADES: Collaborative Approach to Meta-analysis and Review of Animal Data in Experimental Studies
EEVs: Engineered extracellular vesicles
EVs: Extracellular vesicles
FDA: Food and Drug Administration
IS: Ischemic stroke
MCAO: Middle cerebral artery occlusion
MMP9: Matrix metallopeptidase 9
mNSS: Modified neurological severity score
MVs: Microvesicles
NSC: Neural stem cell
PRISMA: Preferred Reporting Items for Systematic Reviews and Meta-Analyses
ROS: Reactive oxygen species
TSP1: Thrombopoietin 1
VEGF: Vascular endothelial growth factor
