# Supplementary material for: Engineered extracellular vesicles for ischemic stroke: a systematic review and meta-analysis of preclinical studies
Source: J Nanobiotechnology. 2023 Oct 31;21:396. doi: 10.1186/s12951-023-02114-8 (PMC10617166; doi:10.1186/s12951-023-02114-8)
Supplement: Supplementary file 1 — Supplementary Material 1 [file 12951_2023_2114_MOESM1_ESM.docx]

**Multimedia Appendix 1.** Details of the search.

**Pubmed**

| **#** | **Search terms** | **Results** |
| --- | --- | --- |
| 1 | "Extracellular Vesicles"[Mesh] | 26,278 |
| 2 | (Extracellular Vesicle[Title/Abstract]) OR (Vesicle, Extracellular[Title/Abstract]) OR (Vesicles, Extracellular[Title/Abstract]) OR (Exovesicles[Title/Abstract]) OR (Exovesicle[Title/Abstract]) OR (Apoptotic Bodies[Title/Abstract]) OR (Apoptotic Body[Title/Abstract]) OR (Bodies, Apoptotic[Title/Abstract]) OR (Body, Apoptotic[Title/Abstract]) OR (Exosomes[Title/Abstract]) OR (Endosomes[Title/Abstract]) OR (Secretory Vesicles[Title/Abstract]) OR (Cell-Derived Microparticles[Title/Abstract]) | 48,909 |
| 3 | #1 OR #2 | 173,181 |
| 4 | "Stroke"[Mesh] | 60,436 |
| 5 | (strokes[Title/Abstract]) OR (cerebrovascular accident[Title/Abstract]) OR (cerebrovascular accidents[Title/Abstract]) OR (cva[Title/Abstract]) OR (cvas[Title/Abstract]) OR (cerebrovascular apoplexy[Title/Abstract]) OR (apoplexy, cerebrovascular[Title/Abstract]) OR (vascular accident, brain[Title/Abstract]) OR (brain vascular accident[Title/Abstract]) OR (brain vascular accidents[Title/Abstract]) OR (vascular accidents, brain[Title/Abstract]) OR (cerebrovascular stroke[Title/Abstract]) OR (cerebrovascular strokes[Title/Abstract]) OR (stroke, cerebrovascular[Title/Abstract]) OR (strokes, cerebrovascular[Title/Abstract]) OR (apoplexy[Title/Abstract]) OR (cerebral stroke[Title/Abstract]) OR (cerebral strokes[Title/Abstract]) OR (stroke, cerebral[Title/Abstract]) OR (strokes, cerebral[Title/Abstract]) OR (stroke, acute[Title/Abstract]) OR (acute stroke[Title/Abstract]) OR (acute strokes[Title/Abstract]) OR (strokes, acute[Title/Abstract]) OR (cerebrovascular accident, acute[Title/Abstract]) OR (acute cerebrovascular accident[Title/Abstract]) OR (acute cerebrovascular accidents[Title/Abstract]) OR (cerebrovascular accidents, acute[Title/Abstract]) | 204,958 |
| 6 | #3 OR #5 | 337 |

**Cochrane Library**

| **#** | **Search terms** | **Results** |
| --- | --- | --- |
| 1 | MeSH descriptor: [Extracellular Vesicles] explode all trees | 207 |
| 2 | (Extracellular Vesicle):ab,ti,kw OR (Vesicle, Extracellular):ab,ti,kw OR (Vesicles, Extracellular):ab,ti,kw OR (Exovesicles):ab,ti,kw OR (Exovesicle):ab,ti,kw OR (Apoptotic Bodies):ab,ti,kw OR (Apoptotic Body):ab,ti,kw OR (Bodies, Apoptotic):ab,ti,kw OR (Body, Apoptotic):ab,ti,kw OR (Exosomes):ab,ti,kw OR (Endosomes):ab,ti,kw OR (Secretory Vesicles):ab,ti,kw OR (Cell-Derived Microparticles):ab,ti,kw | 641 |
| 3 | #1 OR #2 | 656 |
| 4 | MeSH descriptOR: [Stroke] explode all trees | 83,440 |
| 5 | (Strokes):ab,ti,kw OR (cerebrovascular accident):ab,ti,kw OR (cerebrovascular accidents):ab,ti,kw OR (cva):ab,ti,kw OR (cvas):ab,ti,kw OR (cerebrovascular apoplexy):ab,ti,kw OR (apoplexy, cerebrovascular):ab,ti,kw OR (vascular accident, brain):ab,ti,kw OR (brain vascular accident):ab,ti,kw OR (brain vascular accidents):ab,ti,kw OR (vascular accidents, brain):ab,ti,kw OR (cerebrovascular stroke):ab,ti,kw OR (cerebrovascular strokes):ab,ti,kw OR (stroke, cerebrovascular):ab,ti,kw OR (strokes, cerebrovascular):ab,ti,kw OR (apoplexy):ab,ti,kw OR (cerebral stroke):ab,ti,kw OR (cerebral strokes):ab,ti,kw OR (stroke, cerebral):ab,ti,kw OR (strokes, cerebral):ab,ti,kw OR (stroke, acute):ab,ti,kw OR (acute stroke):ab,ti,kw OR (acute strokes):ab,ti,kw OR (strokes, acute):ab,ti,kw OR (cerebrovascular accident, acute):ab,ti,kw OR (acute cerebrovascular accident):ab,ti,kw OR (acute cerebrovascular accidents):ab,ti,kw OR (cerebrovascular accidents, acute):ab,ti,kw | 39,164 |
| 6 | #4 OR #5 | 87,202 |
| 7 | #3 AND #6 | 22 |

**Web of Science**

| **#** | **Search terms** | **Results** |
| --- | --- | --- |
| 1 | AB=(Extracellular Vesicle OR Vesicle, Extracellular OR Vesicles, Extracellular OR Exovesicles OR Exovesicle OR Apoptotic Bodies OR Apoptotic Body OR Bodies, Apoptotic OR Body, Apoptotic OR Exosomes OR Endosomes OR Secretory Vesicles OR Cell-Derived Microparticles) | 97,864 |
| 2 | AB=(Stroke OR Strokes OR Cerebrovascular Accident OR Cerebrovascular Accidents OR CVA (Cerebrovascular Accident) OR CVAs (Cerebrovascular Accident) OR Cerebrovascular Apoplexy OR Apoplexy, Cerebrovascular OR Vascular Accident, Brain OR Brain Vascular Accident OR Brain Vascular Accidents OR Vascular Accidents, Brain OR Cerebrovascular Stroke OR Cerebrovascular Strokes OR Stroke, Cerebrovascular OR Strokes, Cerebrovascular OR Apoplexy OR Cerebral Stroke OR Cerebral Strokes OR Stroke, Cerebral OR Strokes, Cerebral OR Stroke, Acute OR Acute Stroke OR Acute Strokes OR Strokes, Acute OR Cerebrovascular Accident, Acute OR Acute Cerebrovascular Accident OR Acute Cerebrovascular Accidents OR Cerebrovascular Accidents, Acute) | 511,556 |
| 3 | #1 and #2 | 970 |

**Embase via Ovid**

| **#** | **Search terms** | **Results** |
| --- | --- | --- |
| 1 | 'Extracellular Vesicles'/exp | 58,076 |
| 2 | 'Extracellular Vesicle':ab,ti OR 'Vesicle, Extracellular':ab,ti OR 'Vesicles, Extracellular':ab,ti OR 'Exovesicles':ab,ti OR 'Exovesicle':ab,ti OR 'Apoptotic Bodies':ab,ti OR 'Apoptotic Body':ab,ti OR 'Bodies, Apoptotic':ab,ti OR 'Body, Apoptotic':ab,ti OR 'Exosomes':ab,ti OR 'Endosomes':ab,ti OR 'Secretory Vesicles':ab,ti OR 'Cell-Derived Microparticles':ab,ti | 59,359 |
| 3 | #1 OR #2 | 86,691 |
| 4 | 'Stroke'/exp | 428,707 |
| 5 | 'strokes':ab,ti OR 'cerebrovascular accident':ab,ti OR 'cerebrovascular accidents':ab,ti OR 'cva (cerebrovascular accident)':ab,ti OR 'cvas (cerebrovascular accident)':ab,ti OR 'cerebrovascular apoplexy':ab,ti OR 'apoplexy, cerebrovascular':ab,ti OR 'vascular accident, brain':ab,ti OR 'brain vascular accident':ab,ti OR 'brain vascular accidents':ab,ti OR 'vascular accidents, brain':ab,ti OR 'cerebrovascular stroke':ab,ti OR 'cerebrovascular strokes':ab,ti OR 'stroke, cerebrovascular':ab,ti OR 'strokes, cerebrovascular':ab,ti OR 'apoplexy':ab,ti OR 'cerebral stroke':ab,ti OR 'cerebral strokes':ab,ti OR 'stroke, cerebral':ab,ti OR 'strokes, cerebral':ab,ti OR 'stroke, acute':ab,ti OR 'acute stroke':ab,ti OR 'acute strokes':ab,ti OR 'strokes, acute':ab,ti OR 'cerebrovascular accident, acute':ab,ti OR 'acute cerebrovascular accident':ab,ti OR 'acute cerebrovascular accidents':ab,ti OR 'cerebrovascular accidents, acute':ab,ti | 96,320 |
| 6 | #4 OR #5 | 458,661 |
| 7 | #3 and #6 | 767 |

**Scopus**

| **#** | **Search terms** | **Results** |
| --- | --- | --- |
| 1 | ABS(Extracellular Vesicles) OR ABS(Extracellular Vesicle) OR ABS(Vesicle, Extracellular) OR ABS(Vesicles, Extracellular) OR ABS(Exovesicles) OR ABS(Exovesicle) OR ABS(Apoptotic Bodies) OR ABS(Apoptotic Body) OR ABS(Bodies, Apoptotic) OR ABS(Body, Apoptotic) OR ABS(Exosomes) OR ABS(Endosomes) OR ABS(Secretory Vesicles) OR ABS(Cell-Derived Microparticles) | 78,875 |
| 2 | ABS(stroke) OR ABS(strokes) OR ABS(cerebrovascular accident) OR ABS(cerebrovascular accidents) OR ABS(cva) OR ABS(cvas) OR ABS(cerebrovascular apoplexy) OR ABS(apoplexy, cerebrovascular) OR ABS(vascular accident, brain) OR ABS(brain vascular accident) OR ABS(brain vascular accidents) OR ABS(vascular accidents, brain) OR ABS(cerebrovascular stroke) OR ABS(cerebrovascular strokes) OR ABS(stroke, cerebrovascular) OR ABS(strokes, cerebrovascular) OR ABS(apoplexy) OR ABS(cerebral stroke) OR ABS(cerebral strokes) OR ABS(stroke, cerebral) OR ABS(strokes, cerebral) OR ABS(stroke, acute) OR ABS(acute stroke) OR ABS(acute strokes) OR ABS(strokes, acute) OR ABS(cerebrovascular accident, acute) OR ABS(acute cerebrovascular accident) OR ABS(acute cerebrovascular accidents) OR ABS(cerebrovascular accidents, acute) | 393,898 |
| 4 | #1 and #2 | 663 |
